# Hypoglycemic Assessment of Aqueous Leaf Extract of *Moringa oleifera* on Diabetic Wistar Rats

**DOI:** 10.1155/2024/9779021

**Published:** 2024-10-23

**Authors:** Egbujo Ejike Amina, James O. Adisa, Solomon Matthias Gamde, Etinosa Beauty Omoruyi, Habauka M. Kwaambwa, Lamech M. Mwapagha

**Affiliations:** ^1^Department of Medical Laboratory Science, University of Jos, Plateau, Nigeria; ^2^Department of Medical Laboratory Science, Bingham University Karu, New Karu, Nasarawa, Nigeria; ^3^Applied Microbial and Health Biotechnology Institute, Cape Peninsula University of Technology, Cape Town, South Africa; ^4^Department of Biology, Chemistry and Physics, Faculty of Health, Natural Resources and Applied Sciences, Namibia University of Science and Technology, Private Bag 13388, 13 Jackson Kaujeua Street, Windhoek, Namibia

**Keywords:** antioxidants, diabetes, *Moringa oleifera*, toxicity

## Abstract

**Background:** *Moringa oleifera* leaf is used for diabetes due to its pharmacologic effects. Patients with hyperglycemia experience beta cell destruction. However, no research on risk awareness has been done to ascertain its safety. The present study describes the antidiabetic effect of *Moringa oleifera* leaf, such as the protection of pancreatic beta cells and the induction of glycogen synthesis, before addressing the secondary effects of diabetes, such as hepatic and renal toxicity.

**Methods:** Forty-five Wistar rats weighed 160 ± 10 g were divided into nine groups. All animal operations complied with the National Institute of Health (NIH) guidelines for the care and use of laboratory animals as approved by the Animal Ethical Committee, University of Jos. Group I was normal control and Group II was diabetic animals induced with alloxan. Insulin and extract doses of 200, 400, and 800 mg/kg were given to diabetic Groups III-VI. Normal animals in Groups VII–IX were given extract at doses of 200, 400, and 800 mg/kg for 28 days. Tissues were retrieved for biochemical and histological investigations using standard techniques.

**Results:** There was decrease relative body weight of diabetic animals (95.50 ± 5.50) when compared to normal control (142.75 ± 20.08) with increased levels of urea (control 6.13 ± 0.523 and diabetes 29.23 ± 1.267) and creatinine (control 0.70 ± 0.057 and diabetes 2.13 ± 0.185). Histology of the liver and pancreas also points to organ damage due to hyperglycemia. However, oral administration of extract showed antidiabetic effect with protection of pancreatic beta cells and the induction of glycogen synthesis, no glycogen was deposited in the liver, addressing the secondary effects of diabetes, such as hepatic and renal toxicity. Further discovery revealed that extract elevated antioxidant enzyme expression.

**Conclusion:** Leaf extract from *Moringa oleifera* reduces blood sugar and lessens the damage caused by hyperglycemia in the pancreas and liver.

## 1. Introduction

Diabetes mellitus (DM) is a growing public health concern worldwide. According to the World Health Organization (WHO), the number of people living with DM increased dramatically in the last decades, reaching a total number of 422 million cases, with about 1.6 million deaths per year [1]. Diabetes is a chronic systemic disorder with a complex pathogenesis characterized by peripheral insulin resistance, abnormal regulation of hepatic glucose production, and decreased beta-cell function, which ultimately leads to beta-cell failure [2, 3].

Despite the established benefits of integrated multiple risk factor reduction for glycemic control and lipid-lowering therapy inclusive [4–6] controlling diabetes spread remained a global health challenge. From ethnopharmacological surveys, there is abundant epidemiological and clinical evidence of phytotherapeutic products, unveiling the treasure of natural resources for diabetes [7–10].

*Moringa oleifera* (MO) is one of the most popular species of the Moringaceae family, having a significant pharmacological and nutritious value [11]. The leaves are the most prescribed and used part of the tree, being a source of proteins, flavonoids, glycoside, *β*-carotene, and vitamins A, B, and C [11, 12]. MO leaves are used for patients with type II DM [13, 14]. Some people consume MO leaves daily as a nutritional supplement for diabetes [15–17]. The antidiabetic [18], antioxidant [19], antihypertensive [11], and antimicrobial [16] activities of the plant have been established. Although studies have reported the biological and medicinal importance of the various Moringaceae species, no study has documented the effect of MO leaf extract on the biochemical and histopathology of the liver, kidney, and pancreas in diabetes. Apart from specifying the therapeutic objective and suitability as ethnomedicine for treating DM, it is necessary to provide safety instructions for such medications [20]. To this effect, we aimed to determine the hypoglycemic and possible toxicity of MO leaf extract on diabetic Wistar rats.

## 2. Materials and Methods

### 2.1. Plant Material

The leaves of MO were harvested fresh from Langtang Local Government Area and identified at the Forest Herbarium Jos, Nigeria with a voucher number FHJ 238.

### 2.2. Ethical Statement

All animal operations complied with the NIH guidelines for the care and use of laboratory animals as approved by the Animal Ethical Committee, Department of Pharmacology, University of Jos.

### 2.3. Chemicals Used

Alloxan monohydrate (Chemical Co. St. Louis, MO, USA-CAS Number: 2244-11-3) and analytical grade reagents kits (Randox Laboratories Limited, Crumlin, and County Antrim, United Kingdom) for AST, ALT, AP, and Bilirubin/Urea/Creatinine/Albumin (Cat No: AS3804, AL146, AP3802, BR2362, UR3825, CR2336, AB362) were, respectively, purchased from Medicom diagnostics, Jos Nigeria.

### 2.4. Preparation of Extract

Fresh leaves of the plant were air dried and ground into powder. Fifty grams of the powder was weighed and soaked in 100 mL distilled water and allowed to stand for 48 h for extraction [7]. After 48 h, the samples were double filtered using Whatman No. 1 filter paper (LOT. No: 7,229,421). The filtrate was evaporated to dryness at a reduced temperature of 45°C in the water bath.

### 2.5. Determination of Phytochemicals

An initial screening of phytochemical test of the extract was performed according to the standard method described by Shafodino et al. [21, 22]. Various phytochemicals screened for includes presence of phenols using Folin–Ciocalteu test, flavonoids (Alkaline reagent and lead acetate test), saponins (Froth and form test), steroids (acetic anhydride and H_2_SO_4_ test), alkaloids (Mayer's and Wagner's test), tannins (Gelatin test), terpenoids (Salkowski test), carbohydrate (Anthrone test), cardiac glycosides (Keller-Killani test), protein (Ninhydrin test), amino acids (Millon's test), Anthraquinones (Borntrager's test), and polyphenols (Puncal-D test). The extract was further analyzed to estimate its total phenolic content according to the method described by Koncić et al. [23] using Folin–Ciocalteu, with gallic acid (GAE) as the standard. Total flavonoid content was evaluated using rutin (RU) as standard protocol described by Zhishen, Mengcheng, and Jianming [24]. Total alkaloid and anthocyanidin contents were determined using atropine (AP) and vanillin-HCl colorimetric and catechin (CT) as standards described by Ashafa, Sunmonu, and Afolayan [25]. The tests were performed in triplicates to ensure accurate results and were expressed as mean ± SD values.

### 2.6. Animals Used

Forty-five white Wistar rats of both sexes weighing 160 ± 10 g was purchased in the Department of Pharmacology, University of Jos. The animals were housed in cages and maintained under standard husbandry conditions (between 22°C, 12 h light and 12 h dark, and 30%–35% humidity) in the Animal House. Animals were fed *ad libitum* with standard commercial rat feed (Cat No: 0,714,931,256) and clean drinking water. The use of experimental animals was approved by the Animal Ethical Committee, Department of Pharmacology, University of Jos.

### 2.7. Induction of Diabetes

Diabetes was induced according to the procedure described by Das and Sil [26] via intraperitoneal injection of 1% solution of alloxan at 150 mg/kg body weight dissolved in distilled water after fasting the animals overnight. Glucose levels were confirmed on the fourth day using glucometer and test strips. Animals with sugar levels more than 150 mg/dL were diabetic.

### 2.8. Animals Grouping and Dosing

Forty-five animals were randomly assigned into nine groups consisting of five animals each and treated as in Table 1. Insulin and MO extract were administered by oral gavage to the animals once daily for 28 consecutive days.

### 2.9. Sample Collection and Preparation

After the last dose, the animal's body weights were recorded using a weighing balance (CS 200, China). Animals were euthanized and the blood collected in plane tubes were allowed to clot and centrifuged at 3000 rpm for 15 min and hence used for biochemical assays [8]. The liver, kidney, and pancreas were excised via abdominal incision and processed using the paraffin wax method [27].

### 2.10. Biochemical Assays Assessment of Liver Function

The Liver transaminases aspartate aminotransferase (AST), alkaline phosphatase (ALP), alanine aminotransferase (ALT), total protein (TP), albumin (Alb), direct bilirubin (DB), and total bilirubin (TB) were estimated according to the instructions of the manufacturer using assay kits from Randox laboratories, United Kingdom.

### 2.11. Determination of Kidney Function

Serum potassium (K^+^), sodium (Na^+^), chloride (Cl^−^), bicarbonate (HCO_3_), urea (U), and creatinine (Cr) were assessed according to the instructions of the manufacturer using assay kits from Randox laboratories, United Kingdom.

### 2.12. Assessment of Catalase (CAT) Activity

CAT activity was determined according to the method of Sinhna [28]. The hydrogen peroxide (H_2_O_2_) content of the withdrawn sample was determined by taking the absorbance at 570 nm. CAT activity was expressed as l mol H_2_O_2_ consumed/min/mg protein.

### 2.13. Estimation of Superoxide Dismutase (SOD) Activity

SOD activity was determined according to the method of Misra and Fridovich [29] in the homogenates. One unit of SOD activity was defined as the amount of SOD necessary to cause 50% inhibition of the oxidation of adrenaline to adrenochrome during 1 min. The absorbance was read at 400 nm.

### 2.14. Estimation of Glutathione Peroxidase (GPx) Activity

GPx activity estimation was based on the rate of hydrogen peroxide (H_2_O_2)_ consumption as described by Rotruck et al. [30]. GPx activity was determined by extrapolating the concentration of the remaining glutathione (GSH) from a GSH standard curve and expressed as GSH consumed/mg protein.

### 2.15. Histopathology Assessment

The liver, kidney, and pancreas were excised via abdominal incision and were fixed in 10% buffered formalin. Tissues were processed by the paraffin wax method as described by Gamde et al. [31].

### 2.16. Statistical Analysis

The statistical data was analyzed by Student's *t*-test post hoc to compare the significance between experimental groups. Data were expressed as mean and standard deviation (Mean ± SD). All values were considered statistically significant at *p* ≤ 0.05 as compared to the controls.

## 3. Results

### 3.1. Phytochemical Determination

The initial qualitative screening of MO leaf aqueous extract revealed the presence of various polyphenols listed in Table 2.

The yield of the total phenolics, flavonoids, anthocyanidin, and alkaloids in MO aqueous extract are presented in Table 3. Values were analyzed using a one-way ANOVA test to confirm the significant differences of each content at 95% confidence interval. The total phenolics content (26,201 ± 13.5) was observed to be significantly (*p* < 0.05) higher than the total flavonoids (209.0 ± 1.2), followed by total anthocyanidin and alkaloids (74.9 ± 1.03, and 4.01 ± 0.02), respectively, which may be the reason for the extract antidiabetic effect with protection of pancreatic beta cells and the induction of glycogen synthesis.

### 3.2. Effect of Extract on Fasting Blood Glucose

**Figure 1:**
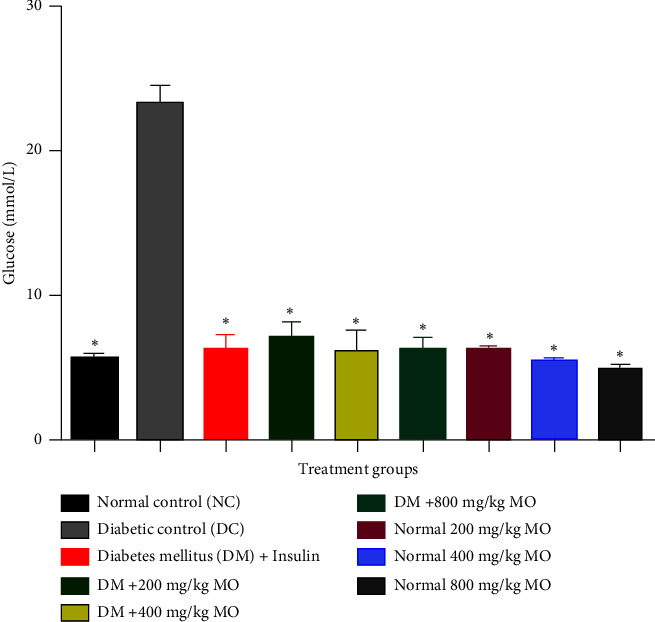
Effect of extract on fasting blood glucose. Effect of MO on fasting blood glucose in alloxan-induced diabetes and control. MO: *Moringa oleifera*; NC: normal control; DC: diabetic control; DM: diabetes mellitus. Values were expressed as mean value ± standard deviation (SD). ⁣^∗^Mean values were significantly different compared with normal control at *p* ≤ 0.05. ^#^Mean values were significantly different compared with diabetic control at *p* ≤ 0.05. With increase in the oral administration of extract concentration led to a significant reduction of fasting blood glucose levels when compared with control.

Administration of alloxan significantly raised the blood glucose levels as compared to the normal control. However, with oral administration of 200, 400, and 800 mg/kg MO extract significantly reduced the fasting blood glucose levels (Figure 1).

### 3.3. Effect of MO Extract on Mean Body Weight

**Figure 2:**
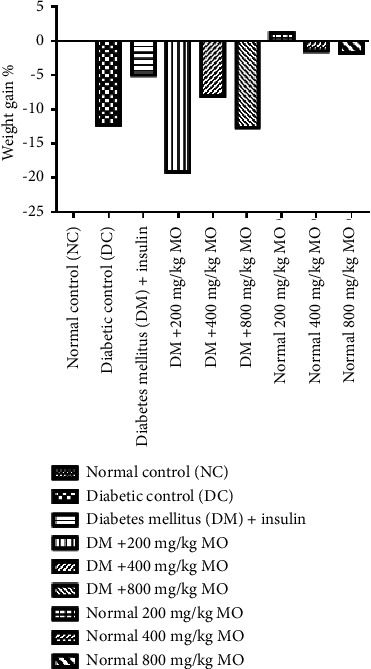
Effect of MO on mean body weight in alloxan-induced diabetes. MO: *Moringa oleifera*; NC: normal control; DC: diabetic control; DM: diabetes mellitus. With increase in the oral administration of extract concentration, there were no significant changes in the body weights when compared with control.

The mean body weights of diabetic control (95.50 ± 0.50) animals significantly decreased when compared to the normal control (142.75 ± 20.08). However, after treatments, the extract did not increase the body weights of diabetic animals when compared to control. Normal animals treated with MO also failed to increase the body weight of the rats except at 200 mg/kg (Figure 2).

### 3.4. Effect of MO Extract on Electrolytes, Urea, and Creatinine

Serum sodium levels in diabetic animals treated with extract 200 mg/kg (*p*=142.67 ± 1.453, *p* < 0.05), 400 mg/kg (*p*=142.67 ± 1.453, *p* < 0.05), and 800 mg/kg (*p*=139.00 ± 0.577, *p* < 0.05) were significantly reduced when compared to diabetic control (*p*=145.33 ± 4.702, *p* < 0.05). In diabetic control animals, the urea (*p*=29.23 ± 1.267, *p* < 0.05) level was significantly increased when compared to normal control (*p*=6.13 ± 0.5230, *p* < 0.05). However, extract significantly reduced urea levels at 200 mg/kg (*p*=6.80 ± 0.568, *p* < 0.05), 400 mg/kg (*p*=7.73 ± 1.637, *p* < 0.05), and 800 mg/kg (*p*=11.66 ± 1.954, *p* < 0.05) when compared to diabetic control animals' urea (*p*=29.23 ± 1.267, *p* < 0.05). Extract at 200 mg/kg (*p*=0.83 ± 0.033, *p* < 0.05), 400 mg/kg (*p*=1.06 ± 0.366, *p* < 0.05), and 800 mg/kg (*p*=1.83 ± 0.523, *p* < 0.05) reduce the creatinine level in diabetic control (*p*=3.13 ± 0.185, *p* < 0.05) level (Table 4).

### 3.5. Effect of MO Extract on Liver Function

Liver enzymes ALP levels in diabetic animals treated with extract at 200 mg/kg (*p*=104.33 ± 5.457, *p* < 0.05), 400 mg/kg (*p*=111.33 ± 10.398, *p* < 0.05), and 800 mg/kg (*p*=128.00 ± 3.786, *p* < 0.05) were significantly reduced when compared to the diabetic animal control (*p*=180.0 ± 9.292, *p* < 0.05). ASP levels in diabetic animals treated with extract at 200 mg/kg (*p*=9.33 ± 1.453, *p* < 0.05), 400 mg/kg (*p*=10.66 ± 1.45, *p* < 0.05), and *8*00 mg/kg (*p*=8.33 ± 0.881, *p* < 0.05) were also significantly reduced when compared to the diabetic animal control (*p*=12.00 ± 0.577, *p* < 0.05). Similarly, serum ALT levels in diabetic animals treated with extract at 200 mg/kg (*p*=5.00 ± 1.000, *p* < 0.05), 400 mg/kg (*p*=5.00 ± 1.528, *p* < 0.05), and 800 mg/kg (*p*=4.33 ± 0.333, *p* < 0.05) were significantly reduced when compared to the diabetic animal control (*p*=7.00 ± 1.000, *p* < 0.05). Furthermore, serum total bilirubin levels in diabetic animals treated with the extract at 200 mg/kg (*p*=6.16 ± 0.441, *p* < 0.05), 400 mg/kg (*p*=9.20 ± 0.907, *p* < 0.05), and 800 mg/kg (*p* < 7.13 ± 0.949, *p* < 0.05) were significantly reduced when compared to diabetic animal control (*p*=14.86 ± 0.902, *p* < 0.05) (Table 5).

### 3.6. Effect of MO Extract on Antioxidant Status

The effect of MO extract on antioxidant enzymes is shown in Table 6. Diabetic animals administered with MO extract showed a dose-dependent increase in the antioxidant enzyme levels. Gpx levels in diabetic animals treated with extract at 200 mg/kg (*p*=10.17 ± 0.802, *p* < 0.05), 400 mg/kg (*p*=10.66 ± 1.037, *p* < 0.05), and 800 mg/kg (*p*=10.68 ± 0.699, *p* < 0.05) showed a dose-dependent increase when compared to the diabetic animal control (*p*=6.45 ± 0.355, *p* < 0.05). SOD levels in diabetic animals treated with extract at 200 mg/kg (*p*=4.57 ± 0.888, *p* < 0.05) and 400 mg/kg (*p*=5.46 ± 0.970, *p* < 0.05) also revealed a dose-dependent increase when compared to the diabetic animal control (*p*=3.56 ± 0.323, *p* < 0.05) except at 800 mg/kg (*p*=3.90 ± 0.773, *p* < 0.05). Similarly, CAT levels were significantly increased in diabetic animals treated with extract 200 mg/kg (*p*=15.79 ± 1.975, *p* < 0.05), 400 mg/kg (*p*=20.45 ± 1.887, *p* < 0.05), and 800 mg/kg (*p*=114.11 ± 1.158, *p* < 0.05) when compared to diabetic animal control (*p*=12.67 ± 0.546, *p* < 0.05).

### 3.7. Histological Assessment

**Figure 3:**
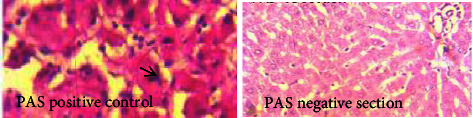
Periodic acid Schiff (PAS) for the demonstration of glycogen deposit in the liver. As controls, PAS + ve means glycogen deposits indicated by purple colour (G) while PAS −ve is negative. With increase in the oral administration of extract concentration, no glycogen deposit was seen when compared with control (PAS. ×400).

**Figure 4:**
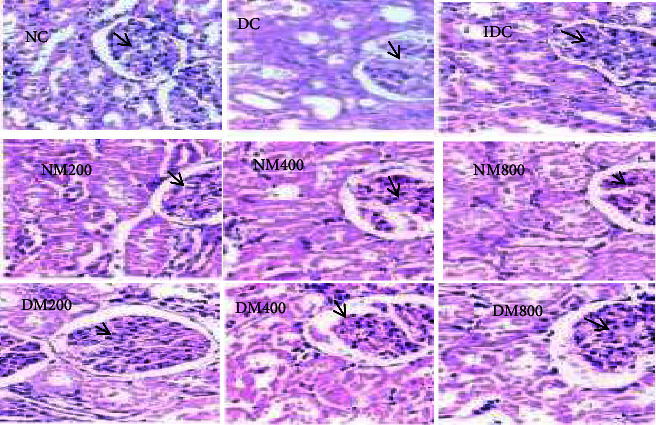
Effect of MO on the kidney in alloxan-induced diabetes. NC: normal control; G: glomeruli; NM: normal animals administered with *Moringa oleifera*; DC: diabetic control; IDC: insulin administered to diabetic animals; DM: diabetes mellitus. With increase in the oral administration of extract concentration, no pathological changes were observed in the kidney histology when compared with control (H & E. ×400).

**Figure 5:**
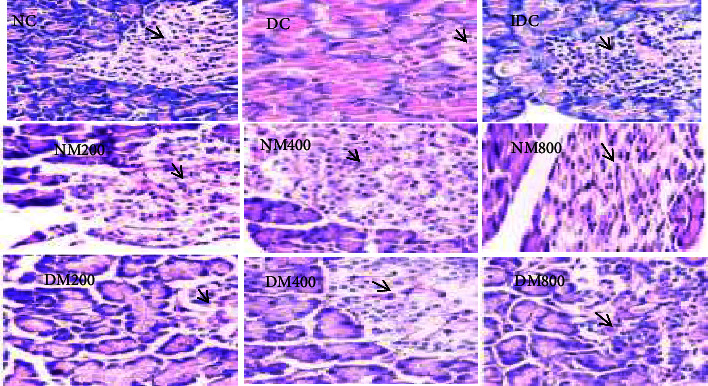
Effect of MO on the pancreas in alloxan-induced diabetes. NC: normal control; IL: islet of Langerhans; NM: normal animals administered with *Moringa oleifera*; DC: diabetic control; IDC: insulin administered to diabetic animals; DM: diabetes mellitus. DC pancreas were atrophied with diminished islet of Langerhans, suggestive of degenerative changes. Increase in the oral administration of extract concentration led to a significant improvement in the parenchyma when compared with control (H & E. ×400).

**Figure 6:**
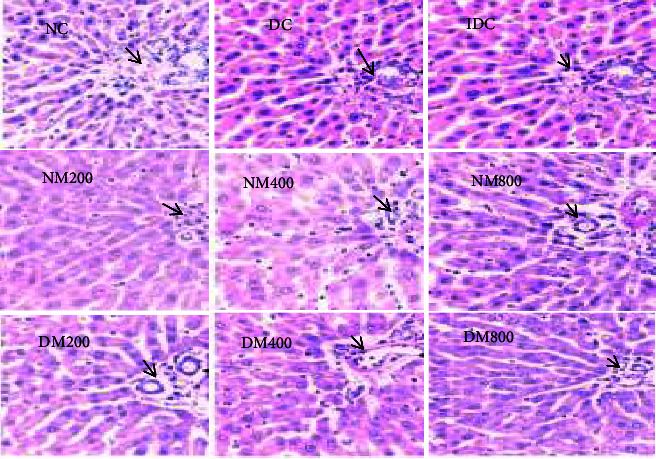
The histopathological effect of MO on liver cells in alloxan-induced diabetes and control. NC: normal control; PT: portal tract; NM: normal animals administered with *Moringa oleifera*; DC: diabetic control; IDC: insulin administered to diabetic animals; DM: diabetes mellitus. DC liver exhibited vacuolations (red arrow) and increased polymorphs (black arrow) suggestive of tissue degeneration. Increase in the oral administration of extract concentration led to a significant improvement in the liver parenchyma when compared with control (H & E stain. ×400).

Histopathological assessment of normal, diabetic, and treated animal's liver, kidney, and pancreas are demonstrated in Figures 3, 4, and 5. Regarding the liver (Figure 6), diabetic animals showed vacuolations with increased polymorphs, suggestive of tissue degeneration. However, diabetic animals treated with insulin and 800 mg/kg MO extract appeared denser, revealing reparation changes. Moreover, no glycogen deposits were found in the liver (Figure 3). In addition, there were no conspicuous histopathological changes documented in the kidneys of diabetic animals compared to normal control (Figure 4). However, the pancreas of diabetic control was atrophied with diminished islet of Langerhans, suggestive of degenerative changes. With increase in the oral administration of extract concentration led to a significant improvement in the parenchyma when compared with control (Figure 5). This is further proved by the up regulation of the antioxidant enzymes which are part of the apoptotic pathway and prevent tissue damage. Moreover, the extracts increased the expression of the antioxidant enzymes which inhibit the expression of antioxidants.

## 4. Discussion

MO possesses a wide range of pharmacological properties, including antioxidant, anti-inflammatory, and antidiabetic effects [32, 33]. Several studies have allotted the therapeutic effect of MO to its combined polyphenols, including flavonoids, alkaloids, phenolics and glucosinolates compounds [34–36]. These components could intermingle with intestinal enzymes by regulating glycemic levels, increase glucose transporters, reduces the synthesis of fatty acids and cholesterol, contributing to the reduction of glucose resistance and blood sugar control [37, 38]. In the study, MO extract significantly reduced the fasting blood glucose levels and protected the pancreatic beta cells and the induction of glycogen synthesis. The antihyperglycemic mechanism could be due to its ability to restore pancreatic function by increasing insulin production sensitivity in peripheral tissues to enhance glucose levels uptake, as well as the inhibition of glucose transporter proteins in cell membranes by flavonoids glycosides [32, 39].

The result showed that alloxan significantly increased blood glucose levels and induced partial to severe damage to the liver, pancreas, and kidney of Wistar rats. This result obtained is similar to the one reported by Hashim and Ayuba [19]. The relative body weights of diabetic animals were significantly decreased compared to normal control. A decrease in the relative body weight suggests organ toxicity due to hyperglycemia [40]. This result is consistent with the report of Aja et al. [7] on body weight loss in diabetic patients due to loss of appetite.

Being an experimental study, the hypoglycemic effect of MO was comparable with the conventional antidiabetic drug insulin. The hypoglycemic effect of MO was in a dose-related manner which agreed with a previous study that observed good glycemic control [15]. This may be due to the extract being readily absorbed in the intraperitoneal cavity and gastrointestinal mucosa. Besides, the body has an integrated antioxidant defense system that scavenges free radicals before they attack tissue cells [41], however, diabetes could cause the body system to fail. It's noteworthy in our result that MO used as starting therapy caused a significant reduction in fasting blood glucose in a short time and confirmed a strong association with oxidative stress markers. Our finding that MO phytochemicals inhibited oxidative damage is consistent with the Al-Bayuomi and Gabr [15] in diabetes patients.

In this perspective, our results exhibited a significant reduction in serum creatinine and urea in the diabetic animals treated with MO. Elevated serum urea and creatinine levels are due to excessive tissue protein breakdown in diabetes [42]. Moreover, electrolyte imbalance is commonly present in patients with type 2 diabetes. In this perspective, our result shows raised serum sodium with increasing fasting blood glucose and a decrease in serum potassium, chloride, and bicarbonate. This agrees with Liamis et al. [43] report but different from Khan et al. [44] which reported decreasing patterns in serum sodium and chloride levels as fasting blood glucose increased [44].

As many authors have suggested, MO significantly reduces liver enzymes as well as total protein, albumin, total, and conjugated bilirubin due to its polyphenolic antioxidant content [45–47]. No glycogen was deposited in the liver sections of the animals treated with MO as compared to the positive control. The propensity of the liver to a normal, suggests the hepatoprotective effect of MO phytochemicals [48, 49]. In addition, diabetes-induced polymorphic cells in the liver, kidney, and pancreatic islets appear to be recovering. The results reported here are consistent with a previous study that reported MO protective effect on pancreatic *β*-cells in diabetes [50] but not consistent with Kandasamy and Ashokkumar [51] study that reported significant changes in the kidney of diabetic rats after treatment. The improved control of blood glucose levels observed in animals treated with insulin and MO can explain the decrease in the production of free radicals. Because of its antidiabetic and antioxidant qualities, the finding of MO's strong hypoglycemic and protective benefits in type 2 diabetes may offer a treatment option for those with the disease. Furthermore, it has been demonstrated that *Moringa* leaf extracts stimulate ROS and act as antioxidants and anticancer agents. Studies have shown that extract elevated antioxidant enzyme expression, which suppresses antioxidant expression [52, 53]. These accounts align with our findings, which indicate increased levels of antioxidant enzymes, which are bio-active substances with antioxidant activity associated with protective effects against chronic degenerative diseases [37].

In conclusion, the hypoglycemic effect of MO leaf extract could be due to its phytochemicals, which are active substances that have been associated with a protective activity against chronic diseases.

## Figures and Tables

**Table 1:** Treatment of animals with insulin and MO extract for 28 days.

Group number	Diabetic or normal animals	Treatment
I	Normal control	Normal control (not treated)
II	Diabetic	150 mg/kg of 1% solution of alloxan
III	Diabetic	Treated daily with insulin
IV	Diabetic	Treated daily with 200 mg/kg MO
V	Diabetic	Treated daily with 400 mg/kg MO
VI	Diabetic	Treated daily with 800 mg/kg MO
VII	Normal (not induced with diabetes)	Treated daily with 200 mg/kg MO
VIII	Normal (not induced with diabetes)	Treated daily with 400 mg/kg MO
IX	Normal (not induced with diabetes)	Treated daily with 800 mg/kg MO

**Table 2:** Qualitative polyphenols present in MO extract.

Polyphenols	Qualitative analysis
Phenolics	+++
Flavonoids	+++
Saponins	++
Steroids	−
Alkaloids	+++
Tannins	+++
Terpenoids	++
Carbohydrate	+
Cardiac glycosides	−
Protein	+
Amino acids	+
Anthraquinones	−
Polyphenols	+++

*Note*: +++ = highly present. ++ = moderately present. + = present. − = not detected.

**Table 3:** Quantification of polyphenols present in MO aqueous extract.

Polyphenols		Quantity
Total phenolics	mg GAE/100 mg dw	26.201 ± 13.5^a^
Total flavonoids	mg RU/100 mg dw	209.0 ± 1.2^b^
Total anthocyanidin	mg CT/100 mg dw	74.9 ± 1.03^c^
Total alkaloids	mg AP/100 mg dw	4.01 ± 0.02^d^

*Note*: Data represent three replicates (*n* = 3) mean value ± SD. Superscript a, b, c, d represent significant differences (*p* < 0.05) of each quantification.

Abbreviations: AP, atropine equivalent; CT, catechin equivalent; dw, dry weight; GAE, gallic acid equivalent; RU, rutin equivalent.

**Table 4:** Effect of MO on electrolytes, urea, and creatinine in alloxan-induced diabetes.

Group	Na^+^ (mmol/L)	K^+^ (mmol/L)	Cl^−^ (mmol/L)	HCO_3_^−^ (mmol/L)	Urea (mmol/L)	Creatinine (mg/dL)
NC	142.33 ± 3.930	5.03 ± 0.120	99.66 ± 1.202	25.33 ± 0.666	6.13 ± 0.523	0.80 ± 0.057
DC	145.33 ± 4.702⁣^∗^	4.13 ± 0.202⁣^∗^	98.66 ± 0.881⁣^∗^	21.00 ± 1.155^#^	29.23 ± 1.267⁣^∗^	3.13 ± 0.185⁣^∗^
DM + INSULIN	139.00 ± 1.732⁣^∗^^#^	3.86 ± 0.233⁣^∗^^#^	100.33 ± 1.764⁣^∗^^#^	19.66 ± 1.202⁣^∗^^#^	10.06 ± 1.214⁣^∗^^#^	1.63 ± 0.466⁣^∗^^#^
DM + 200 mg/kg MO	142.67 ± 1.453⁣^∗^^#^	4.23 ± 0.260⁣^∗^^#^	98.33 ± 0.881⁣^∗^^#^	21.33 ± 1.453⁣^∗^^#^	6.80 ± 0.568⁣^∗^^#^	0.83 ± 0.033⁣^∗^^#^
DM + 400 mg/kg MO	142.67 ± 1.453⁣^∗^^#^	4.03 ± 0.202⁣^∗^^#^	100.33 ± 0.881⁣^∗^^#^	20.33 ± 0.881⁣^∗^^#^	7.73 ± 1.637⁣^∗^^#^	1.06 ± 0.366⁣^∗^^#^
DM + 800 mg/kg MO	139.00 ± 0.577⁣^∗^^#^	4.30 ± 0.200⁣^∗^^#^	98.00 ± 0.577⁣^∗^^#^	22.00 ± 1.000⁣^∗^^#^	11.66 ± 1.954⁣^∗^^#^	1.83 ± 0.523⁣^∗^^z^
N + 200 mg/kg MO	138.00 ± 1.732⁣^∗^^#^	4.50 ± 0.115⁣^∗^^#^	98.66 ± 0.881⁣^∗^	23.00 ± 0.577⁣^∗^^#^	4.33 ± 0.145⁣^∗^^#^	0.79 ± 0.006⁣^∗^^#^
N + 400 mg/kg MO	141.67 ± 4.410⁣^∗^^#^	4.30 ± 0.264⁣^∗^^#^	98.66 ± 1.764⁣^∗^	22.33 ± 1.202⁣^∗^^#^	3.90 ± 0.208⁣^∗^^#^	0.75 ± 0.028⁣^∗^^#^
N + 800 mg/kg MO	136.67 ± 0.333⁣^∗^^#^	3.76 ± 0.176⁣^∗^^#^	96.00 ± 0.577⁣^∗^^#^	19.33 ± 0.881⁣^∗^^#^	4.36 ± 0.466⁣^∗^^#^	0.76 ± 0.088⁣^∗^^#^

*Note*: Values were expressed as mean value ± standard deviation (SD).

Abbreviations: Cl^−^: chloride, DC: diabetic control; DM: diabetes mellitus; HCO_3_: bicarbonate; K^+^: serum potassium; MO: *Moringa oleifera*; NC: normal control; N: normal animal; Na^+^: sodium.

^∗^Mean values were significantly different compared with normal control at *p* ≤ 0.05. ^#^Mean values were significantly different compared with diabetic control at *p* ≤ 0.05. With increase in extract concentration led to a significant reduction of electrolytes, urea, and creatinine levels when compared with control.

**Table 5:** Effect of MO on liver function parameter in alloxan-induced diabetes.

Group	ALP (µ/L)	ALT (µ/L)	AST (µ/L)	TP (g/L)	A (g/L)	TB (µmol/L)	CB (µmol/L)
NC	167.0 ± 10.066	3.66 ± 0.333	8.66 ± 0.881	6.70 ± 0.208	4.03 ± 0.240	3.43 ± 1.335	4.03 ± 1.033
DC	180.0 ± 9.292⁣^∗^	7.00 ± 1.000⁣^∗^	12.00 ± 0.577⁣^∗^	6.83 ± 0.375⁣^∗^	4.26 ± 0.470⁣^∗^	14.86 ± 0.902⁣^∗^	3.63 ± 0.233⁣^∗^
DM + INSULIN	113.00 ± 5.292⁣^∗^^#^	5.00 ± 1.000⁣^∗^^#^	9.33 ± 1.453⁣^∗^^#^	7.26 ± 0.318⁣^∗^^#^	4.66 ± 0.318⁣^∗^^#^	5.86 ± 0.895⁣^∗^^#^	5.10 ± 3.002⁣^∗^^#^
DM + 200 mg/kg MO	104.33 ± 5.457⁣^∗^^#^	5.00 ± 1.528⁣^∗^^#^	10.66 ± 1.45⁣^∗^^#^	6.63 ± 0.145⁣^∗^^#^	4.03 ± 0.120^#^	6.16 ± 0.441⁣^∗^^#^	2.00 ± 0.264⁣^∗^^#^
DM + 400 mg/kg MO	111.33 ± 10.398⁣^∗^^#^	4.33 ± 0.333⁣^∗^^#^	8.33 ± 0.881⁣^∗^^#^	6.00 ± 0.057⁣^∗^^#^	3.36 ± 0.088⁣^∗^^#^	9.20 ± 0.907⁣^∗^^#^	2.66 ± 0.145⁣^∗^^#^
DM + 800 mg/kg MO	128.00 ± 3.786⁣^∗^^#^	5.33 ± 1.333⁣^∗^^#^	8.66 ± 1.764^#^	6.83 ± 0.202⁣^∗^	4.53 ± 0.272⁣^∗^^#^	7.13 ± 0.949⁣^∗^^#^	2.43 ± 0.338⁣^∗^^#^
N + 200 mg/kg MO	102.67 ± 14.099⁣^∗^^#^	3.66 ± 0.333^#^	7.33 ± 0.333⁣^∗^^#^	6.43 ± 0.352⁣^∗^^#^	3.53 ± 0.393⁣^∗^^#^	6.60 ± 0.208⁣^∗^^#^	2.86 ± 0.176⁣^∗^^#^
N + 400 mg/kg MO	110.67 ± 6.692⁣^∗^^#^	4.33 ± 0.333⁣^∗^^#^	8.66 ± 0.333^#^	6.63 ± 0.088⁣^∗^^#^	4.10 ± 0.416⁣^∗^^#^	5.33 ± 0.284⁣^∗^^#^	2.10 ± 0.057∗^#^
N + 800 mg/kg MO	104.67 ± 18.442⁣^∗^^#^	5.66 ± 1.202⁣^∗^^#^	9.00 ± 0.577⁣^∗^^#^	6.06 ± 0.120⁣^∗^^#^	3.46 ± 0.120⁣^∗^^#^	5.60 ± 0.208⁣^∗^^#^	2.66 ± 0.120⁣^∗^^#^

*Note*: Effect of MO on liver function parameter in alloxan-induced diabetes and control. Values were expressed as mean value ± standard deviation (SD).

Abbreviations: ALP: alkaline phosphatase; ALT: alanine aminotransferase; A: albumin; AST: aspartate aminotransferase; CB: conjugated bilirubin; DC: diabetic control; DM: diabetes mellitus; MO: *Moringa oleifera*; N: normal animal; NC: normal control; TP: total protein; and TB: total bilirubin.

^∗^Mean values were significantly different compared with normal control at *p* ≤ 0.05. ^#^Mean values were significantly different compared with diabetic control at ≤ 0.05. Increase in extract concentration led to a significant reduction in aspartate aminotransferase, alkaline phosphatase, alanine aminotransferase, total protein, albumin, direct bilirubin, and total bilirubin levels when compared with control.

**Table 6:** Effect of MO on serum antioxidant status in alloxan-induced diabetes.

Group	Gpx (µmole/min/mg protein)	CAT (µmole/H_2_O_2_/min/mg/protein)	SOD (µmole/SOD/min/mg protein)
NC	3.95 ± 0.223	6.67 ± 0.283	2.32 ± 0.256
DC	6.45 ± 0.355⁣^∗^	12.67 ± 0.546⁣^∗^	3.56 ± 0.323⁣^∗^
DM + INSULIN	7.22 ± 0.357⁣^∗^^#^	13.43 ± 0.597⁣^∗^^#^	3.43 ± 0.299⁣^∗^^#c^
DM + 200 mg/kg MO	10.17 ± 0.802⁣^∗^^#^	15.79 ± 1.975⁣^∗^^#^	4.57 ± 0.888⁣^∗^^#^
DM + 400 mg/kg MO	10.66 ± 1.037⁣^∗^^#^	20.45 ± 1.887⁣^∗^^#^	5.46 ± 0.970⁣^∗^^#^
DM + 800 mg/kg MO	10.68 ± 0.699⁣^∗^^#^	14.11 ± 1.158⁣^∗^^#^	3.90 ± 0.773⁣^∗^^#^
N + 200 mg/kg MO	5.96 ± 0.433⁣^∗^^#^	17.72 ± 0.450⁣^∗^^#^	5.45 ± 0.082⁣^∗^^#^
N + 400 mg/kg MO	6.75 ± 0.474⁣^∗^^#^	20.55 ± 1.884⁣^∗^^#^	6.94 ± 0.104⁣^∗^^#^
N + 800 mg/kg MO	7.82 ± 0.166⁣^∗^^#^	18.07 ± 1.247⁣^∗^^#^	5.44 ± 0.031⁣^∗^^#^

*Note*: Values were expressed as mean value ± standard deviation (SD).

Abbreviations: CAT: catalase; DC: diabetic control; DM: diabetes mellitus; Gpx: glutathione peroxidase; MO: Moringa oleifera; N: normal animals; NC: normal control; and SOD: superoxide dismutase.

^∗^Mean values were significantly different compared with normal control at *p* ≤ 0.05. ^#^Mean values were significantly different compared with diabetic control at *p* ≤ 0.05. Increase in extract concentration led to a significant increase in glutathione peroxidase, catalase, and superoxide dismutase levels when compared with control.

## Data Availability

The data that support the findings of this study are available from the corresponding author upon reasonable request.
